# Measures of retinal health successfully capture risk for Alzheimer's disease and related dementias at midlife

**DOI:** 10.1177/13872877251321114

**Published:** 2025-03-03

**Authors:** Ashleigh Barrett-Young, Aaron Reuben, Avshalom Caspi, Kirsten Cheyne, David Ireland, Jesse Kokaua, Sandhya Ramrakha, Yih-Chung Tham, Reremoana Theodore, Graham Wilson, Tien Yin Wong, Terrie Moffitt

**Affiliations:** 1 Department of Psychology, University of Otago, Dunedin, New Zealand; 2 Department of Psychology, University of Virginia, Charlottesville, VA, USA; 3 Department of Psychology & Neuroscience, Duke University, Durham, NC, USA; 4 Center for Genomic and Computational Biology, Duke University, Durham, NC, USA; 5 Social, Genetic, and Developmental Psychiatry Centre, Institute of Psychiatry, Psychology, & Neuroscience, King's College London, London, UK; 6 PROMENTA, Department of Psychology, University of Oslo, Norway; 7 Department of Psychiatry and Behavioral Sciences, Duke University, Durham, NC, USA; 8 Va’a o Tautai—Centre for Pacific Health, University of Otago, Dunedin, New Zealand; 9 Singapore National Eye Centre, Singapore Eye Research Institute, Singapore; 10 Centre for Innovation and Precision Eye Health, Department of Ophthalmology, Yong Loo Lin School of Medicine, National University of Singapore, Singapore; 11 Ophthalmology and Visual Science Academic Clinical Program, Duke-NUS Medical School, Singapore; 12 Dunedin School of Medicine, University of Otago, Dunedin, New Zealand; 13 School of Clinical Medicine, Beijing Tsinghua Changgung Hospital, Tsinghua Medicine, Tsinghua University, Beijing, China

**Keywords:** Alzheimer's disease, dementia, fundus oculi, optical coherence tomography, retina, retinal ganglion cells, retinal vessels

## Abstract

**Background:**

Identification of at-risk individuals who would benefit from early intervention for Alzheimer's disease and related dementias (ADRD) is critical as new treatments are developed. Measures of retinal health could offer accessible and low-cost indication of pre-morbid disease risk, but their association with ADRD risk is unknown.

**Objective:**

To determine whether midlife retinal neuronal and microvascular measures are associated with ADRD risk-index scores and individual domains of ADRD risk.

**Methods:**

Data were from the Dunedin Multidisciplinary Health and Development Study, a population-representative longitudinal New Zealand-based birth cohort study. 94.1% (N = 938) of living Study members were seen at age 45 (2017–2019). Retinal neuronal (retinal nerve fiber layer (RNFL) and ganglion cell–inner plexiform layer (GC-IPL)) and microvascular (arterioles and venules) measures were used as predictors. Outcome measures were four top ADRD risk indexes (CAIDE, LIBRA, Lancet, and ADU-ADRI), and a comprehensive midlife ADRD risk index, the DunedinARB.

**Results:**

Poorer retinal microvascular health (narrower arterioles and wider venules) was associated with greater ADRD risk (βs = 0.16–0.31; *p*s < 0.001). Thinner RNFL was modestly associated with higher ADRD risk (βs = 0.05–0.08; *p*s = 0.02–0.13). Follow-up tests of distinct domains of ADRD risk indicated that while RNFL associations reflected cardiometabolic risk only, microvascular measures were associated with diverse ADRD risk factors.

**Conclusions:**

Measures of retinal health, particularly microvascular measures, successfully capture ADRD risk across several domains of known risk factors, even at the young midlife age of 45 years. Retinal microvascular imaging may be an accessible, scalable, and relatively low-cost method of assessing ADRD risk among middle-aged adults.

## Introduction

Progress in the pharmacological and multimodal treatment of Alzheimer's disease and related dementias (ADRD) has led to an increasing imperative to successfully identify individuals who may benefit most from early intervention.^1^ Checklist risk indexes and preclinical disease biomarkers hold the most promise for identifying at-risk individuals before pathology (e.g., neural atrophy) has advanced too far for successful treatment. In this regard, the best risk markers will be usable at-scale among middle-aged (40+) and older (60+) populations, when the earliest pathological processes of ADRD may be underway but not yet manifest as cognitive symptoms or substantial irreversible neurodegeneration.

The retina has been proposed as the site of potentially scalable biomarkers for early ADRD detection as it is the only part of the central nervous system that can be observed directly.^2,3^ The retina is readily imaged using non-invasive, cost-effective, and widely available technology, including optical coherence tomography (OCT) and retinal fundus photography, two imaging modalities available at most eye clinics and from many retail optometrists. Thus, there is a pressing need to better understand how the retina reflects brain health, particularly neurodegeneration, in midlife.^4^

In the *US National Plan to Address Alzheimer's Disease*, ADRDs include Alzheimer's disease and frontotemporal degeneration (FTD), Lewy body dementia (LBD), vascular contributions to cognitive impairment and dementia (VCID), and multiple-etiology dementias (MED).^5^ Retina-based biomarkers have been associated with each of these conditions^6–10^ as well as early cognitive decline and mild cognitive impairment (MCI) at older ages.^8,11–14^ However, their utility as preclinical biomarkers for ADRDs in a middle-aged population remains under-characterized, and it is not yet clear to what extent variation in retinal measures in midlife characterizes individuals otherwise known to be at higher or lower risk for later ADRDs.

There are two classes of retina-based biomarkers that may have utility for ADRD risk prediction: (1) neuronal measures, and (2) microvascular measures. Investigation of each may provide insight into the mechanisms by which the retina reflects the integrity of the rest of the brain. Neuronal measures, encompassing the ganglion cell–inner plexiform layer (GC-IPL), and retinal nerve fiber layer (RNFL), are comprised of the somata, dendrites, and axons of retinal ganglion cells, which form the optic nerve and synapse directly on the brain.^2,3^ Through this direct connection, it is believed that the retina may experience and reflect ongoing pathophysiology elsewhere in the brain; particularly among degraded neurons, as in Alzheimer's disease, by the presence of amyloid plaques and tau neurofibrillary tangles. Thus, the thinning of retinal neuronal layers is thought to reflect atrophy in the brain.^4,13^ Microvascular measures encompass the retinal microvasculature—the arterioles and venules of the retina—which are believed to reflect the integrity of the overall cardiovascular system of the body (including the cerebrovasculature), which is implicated in the pathology of ADRD and, in particular, vascular dementias.^15,16^

Clarity about the associations of neuronal and microvascular retina measures with known ADRD risk factors (e.g., cardiometabolic, inflammatory, etc.) will aid in our understanding of the interplay of retinal biomarkers with mechanistic ADRD risk. Here we report the first investigation of the extent to which measures of retinal neuronal thickness (RNFL and GC-IPL) and microvasculature (arteriole and venule caliber) identify individuals at higher ADRD risk at midlife, in a population-representative birth cohort followed to age 45 years (the New Zealand-based Dunedin Study). We hypothesize that poorer retinal neuronal parameters (thinner neuronal layers) and worse microvascular parameters (narrower arterioles and wider venules) will be associated with greater ADRD risk even at the relatively young age of 45 years.

## Methods

### Study design and population

Participants were members of the Dunedin Multidisciplinary Health and Development Study, a population-representative birth cohort (N = 1037; 91% of eligible births, 51.6% male) born between 1 April 1972 and 31 March 1973 in New Zealand. The cohort represents the full range of socioeconomic status in the general population of New Zealand and is predominantly New Zealand European/Pākehā. The study design and participant characteristics have been described extensively elsewhere.^17^ Assessments were carried out at birth and ages 3, 5, 7, 9, 11, 13, 15, 18, 21, 26, 32, 38, and most recently at age 45 (2016–2019), when 94% of the 997 living Study members participated. The Dunedin Study was approved by the Health and Disability Ethics Committee, Ministry of Health, New Zealand. Written informed consent was obtained from all Study members. Study members were offered a small reimbursement for their time and travel expenses.

### Measures of retinal health

#### Neuronal measures

Neuronal retinal measures were gathered via OCT at age 45 years. OCT scans were performed in the morning by trained technicians using a spectral domain OCT machine (Cirrus HD-OCT, model 5000; Carl Zeiss Meditec). Two scans were taken of each eye, one centered on the optic nerve head (200 × 200) and one centered on the macula (512 × 128) to produce measures of mean peripapillary RNFL thickness and mean macular GC-IPL thickness. The pupils were not dilated pharmacologically prior to scanning. All scans were checked for quality by trained graders and reviewed by ophthalmologists for diseases affecting the retina. Scans were removed from the final dataset due to image quality (e.g., signal strength below 6, scan not correctly positioned, or image artefacts) resulting in n = 31 RNFL data removed and n = 35 GC-IPL data removed from the dataset. 14 Study members were removed due to diseases affecting the retina (glaucoma, multiple sclerosis, retinitis pigmentosa, brain tumors, diabetic laser pan-retinal photocoagulation, and an anomalous optic nerve head). When quality data from only one eye were available, that eye was used; otherwise, final measurements reflect a mean of measurements from both eyes.

#### Microvasculature measures

Retinal microvasculature measures of arteriolar and venular caliber (the size of the lumen or internal space of the vessel) were measured via digital fundus images taken at age 45 years via a Canon CR-1 camera with a 20D single-lens reflex backing. Two photographs were taken of each eye, one centered on the optic disk and another on the fovea, after participants had adapted to dark conditions for five minutes. Both left and right eyes were photographed, and an average taken for use in study analyses. Retinal photographs were graded at the Singapore Eye Research Institute, National University of Singapore, using semi-automated computer software (Singapore I Vessel Assessment (SIVA) Version 3.0). Trained graders, who were blind to participant characteristics, evaluated the SIVA automated measurements and performed manual interventions to correct the software as needed.^18^ Microvessel calibers were based on the six largest arterioles and venules passing through a region located 0.50 to 2.00 disk-diameters from the optic disk margin, a distance which provides a good representation of overall retinal vascular structure.^18^ These measurements were summarized as central retinal artery equivalent (CRAE/“arterioles”) and central retinal vein equivalent (CRVE/“venules”), using the revised Knudtson-Parr-Hubbard formula.^18,19^

### Measures of midlife risk for Alzheimer's disease and related dementias (ADRD risk indexes)

Midlife risk for later ADRD was measured via five top ADRD risk indexes suitable for use in midlife, all generated at age 45 years from data collected across the participants’ lives and at age 45. These included four external indexes first generated and validated in other cohorts and one comprehensive midlife risk index first generated in the Dunedin Study. The five indexes were:

1. The Cardiovascular Risk Factors, Aging, and Incidence of Dementia (CAIDE) index;^20^
2. The LIfestyle for BRAin health (LIBRA) index;^21^
3. The Australian National University Alzheimer's Disease Risk Index (ANU-ADRI);^22^ and
4. Modifiable risk factors selected by the Lancet Commission on Dementia (Lancet);^23^ and
5. A comprehensive midlife index, the Dunedin ADRD Risk Benchmark (DunedinARB), comprised of 48 putative ADRD risk indicators organized into 10 conceptually distinct risk domains.^24^

These indexes are checklists that aggregate and empirically weight risk factors known to increase dementia risk because they are either robustly predictive of dementia (e.g., hearing impairment, a history of depression) or are believed to mechanistically precipitate or enhance disease processes (e.g., *APOE* status, heart disease). Supplemental Material (Appendix 1, Supplemental Figure 1, Supplemental Tables 1 and 2), describes the risk factors present in each index as well as the cut-offs and weighting used to generate final risk-index scores. Measures of retinal arteriolar and venular caliber were excluded from the DunedinARB to enable their use as predictor variables in this study. Risk index scores were generated for all 938 Study members who attended the age-45 assessment phase, as previously described.^24^

Data analysis proceeded in two stages. First, multivariable linear regression models were used to test the association of the retinal health measures (independent variables) with the five global ADRD risk indexes (dependent variables), each examined independently. Second, multivariable linear regression models were used to test the association of the retinal health measures (independent variables) with the 10 unique domains of risk comprising the DunedinARB (dependent variables), each examined independently. Supplementary analyses tested associations between retinal health measures and 9 of the domains of risk while statistically controlling for cardiometabolic risk, the tenth domain. Data were checked and met assumptions of multivariate regressions; no outliers were excluded. All models were adjusted for sex; models with RNFL and GC-IPL were additionally adjusted for retinal axial length. As is standard in tests of retinal microvasculature, retinal artery and vein measures were entered into models together as they are known to covary.^18^

The full project premise and analysis plan was preregistered and is available online (https://tinyurl.com/55rtb3j7). Statistical analysis was performed in Stata SE (v17.0) between August 2023 and August 2024. All *p* values were two-sided, alpha set to 0.05. We additionally applied the Benjamini-Hochberg FDR correction for multiple comparisons where relevant.^25^ Analyses were checked for reproducibility by an independent statistician, who recreated the output using the manuscript and an unaltered copy of the dataset. Although retinal measures are known to change with age,^26^ all participants were the same chronological age at the time of assessment and thus age was not included as a covariate in any models. STROBE reporting guidelines were followed.^27^

## Results

938 (50.5% male) Study members attended the midlife, age-45 assessment wave of the Dunedin Study (94.1% of the original birth-cohort members alive at age 45, Table 1). Supplemental Material (Appendix 2) presents an attrition analysis documenting that Study members who participated in the age-45 assessment did not differ from those who did not. The analytic sample (N = 857 to 883) included all Study members with present age-45 retinal health data and no diseases affecting the retina (e.g., glaucoma).

**Table 1.** Descriptive statistics on the complete assessed cohort at age 45 years (N = 938).

Variable	N	Mean	Std. Dev.	Min	Max
Sex					
Male	474				
Female	464				
Retinal health measures					
Mean retinal nerve fiber layer (µm)	861	92.85	9.31	63.00	126.00
Mean ganglion cell-inner plexiform layer (µm)	857	82.89	5.85	62.00	103.00
Central retinal artery equivalent (µm)	883	139.07	10.42	106.83	179.51
Central retinal vein equivalent (µm)	883	193.82	14.56	148.36	250.39
ADRD Risk Indexes					
CAIDE	938	3.42	2.61	0.00	12.00
LIBRA	938	−0.01	2.93	−5.90	9.00
Lancet	938	4.91	2.83	0.00	13.40
ANU-ADRI	938	2.42	5.81	−10.00	23.00
DunedinARB	938	0.00	4.86	−9.63	19.90

ADRD: Alzheimer's disease and related dementias; CAIDE: Cardiovascular Risk Factors, Aging, and Incidence of Dementia index; LIBRA: LIfestyle for BRAin health index; Lancet: Risks selected by the Lancet Commission on Dementia; ANU-ADRI: Australian National University Alzheimer's Disease Risk Index; DunedinARB: Dunedin ADRD Risk Benchmark.

### Do measures of retinal health capture ADRD risk at age 45 years?

**Figure 1.**
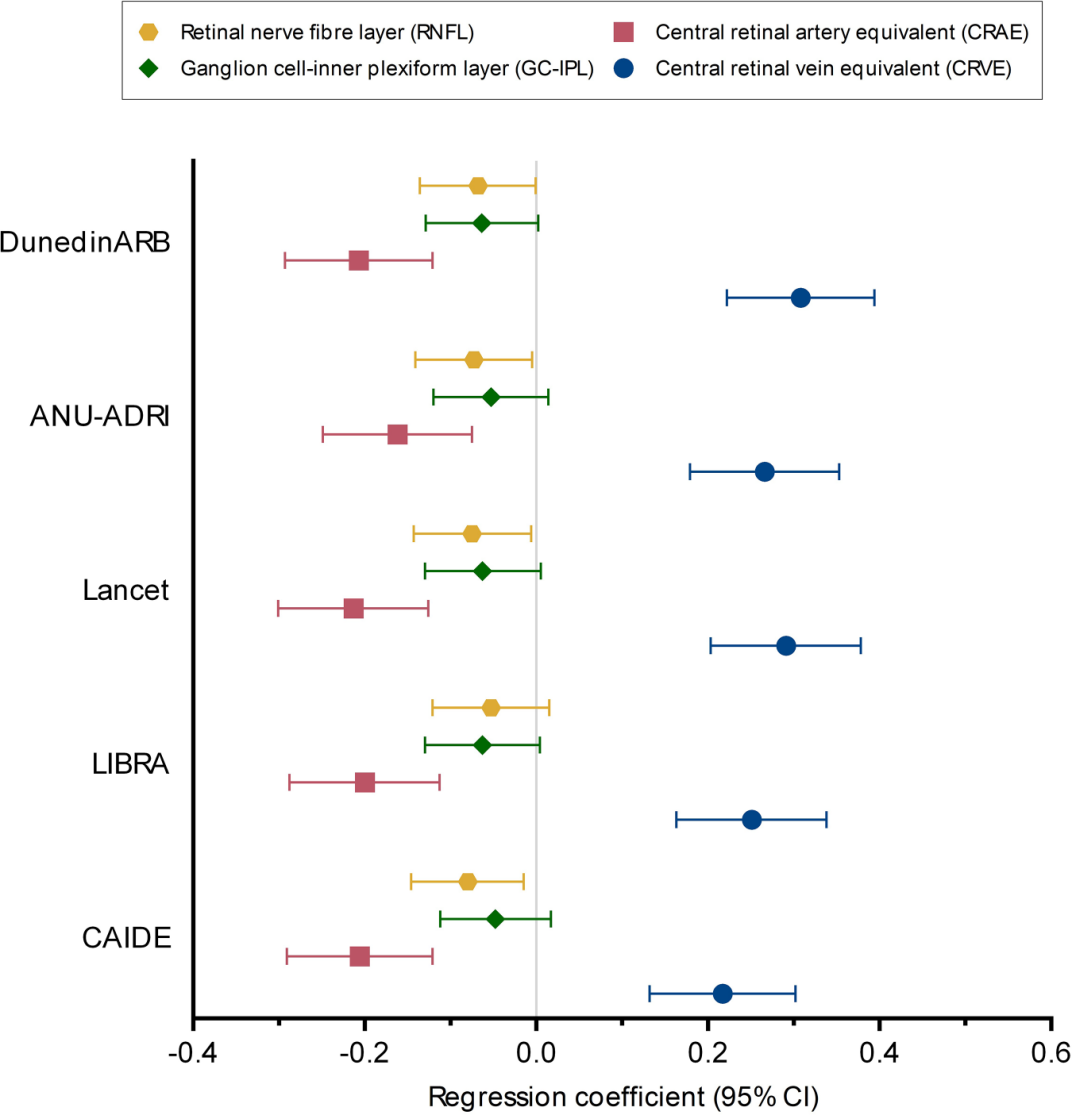
Association of retinal health measures with midlife scores on the 5 ADRD risk indexes.

Evaluation of the measures of retinal neuronal integrity (RNFL and GC-IPL) produced mixed findings (Figure 1). Study members with thinner RNFL were statistically significantly more likely to be classified as having higher midlife ADRD risk, although the associations were modest. For every 10 µm thinner RNFL layer, Study members had 0.06–0.09 standard deviations higher risk-index scores (CAIDE β = −0.08 [95%CI −0.15,−0.02], *p *= 0.02, *R*^2 ^= 0.10; Lancet β = −0.08 [−0.14,−0.01], *p *= 0.03, *R*^2^ = 0.02; ANU-ADRI β = −0.07 [−0.14,-0.01], *p *= 0.04, *R*^2 ^= 0.03; DunedinARB β = −0.07 [−0.14,−0.001], *p *= 0.048, *R*^2 ^= 0.02; LIBRA β = −0.05 [−0.12,0.02], *p *= 0.13, *R*^2 ^= 0.03). Only the association between RNFL and CAIDE remained significant after FDR correction. Associations of the GC-IPL with the ADRD risk indexes were more modest (βs of −0.04 to −0.07) and did not achieve statistical significance.

Figure presents standardized regression coefficients (βs) and 95% confidence intervals for each retinal measure. Models with RNFL and GC-IPL were adjusted for sex and axial length. CRAE and CRVE were included in the same model and adjusted for sex. RNFL N* *= 861, GC-IPL N* *= 857, CRVE/CRAE N* *= 883. DunedinARB: Dunedin ADRD Risk Benchmark; ANU-ADRI: Australian National University Alzheimer's Disease Risk Index; Lancet: risks selected by the Lancet Commission on Dementia; LIBRA: LIfestyle for BRAin health index; CAIDE: Cardiovascular Risk Factors, Aging, and Incidence of Dementia index.

Evaluation of the measures of retinal microvasculature (arterioles and venules) produced consistent and significant findings. Study members with smaller retinal arterioles and wider retinal venules were statistically significantly more likely to be classified as having higher midlife ADRD risk. For every 10 µm decrease in mean arteriole width, Study members had 0.16–0.20 standard deviations higher risk-index scores (Lancet β = −0.21 [95%CI −0.30,−0.13], *p *< 0.001, *R*^2 ^= 0.06; DunedinARB β = −0.21 [−0.29,−0.12], *p *< 0.001, *R*^2 ^= 0.07; CAIDE β = −0.21 [−0.29, −0.12], *p *< 0.001, *R*^2 ^= 0.11; LIBRA β = −0.20 [−0.29,−0.11], *p *< 0.001, *R*^2 ^= 0.06; ANU-ADRI β = −0.16 [−0.25,−0.08], *p *< 0.001, *R*^2 ^= 0.05). All these associations remained significant after FDR correction. For every 10 µm increase in mean venule width, Study members had 0.15–0.20 standard deviations higher risk-index scores (DunedinARB β = 0.31 [95%CI 0.22,0.39], *p *< 0.001, *R*^2 ^= 0.07; Lancet β = 0.29 [0.20,0.38], *p *< 0.001, *R*^2 ^= 0.06; ANU-ADRI β = 0.27 [0.18,0.35], *p *< 0.001, *R*^2 ^= 0.05; LIBRA β = 0.25 [0.16,0.34], *p *< 0.001, *R*^2 ^= 0.06; CAIDE β = 0.22 [0.13,0.30], *p *< 0.001, *R*^2 ^= 0.11). All these associations remained significant after FDR correction.

**Figure 2.**
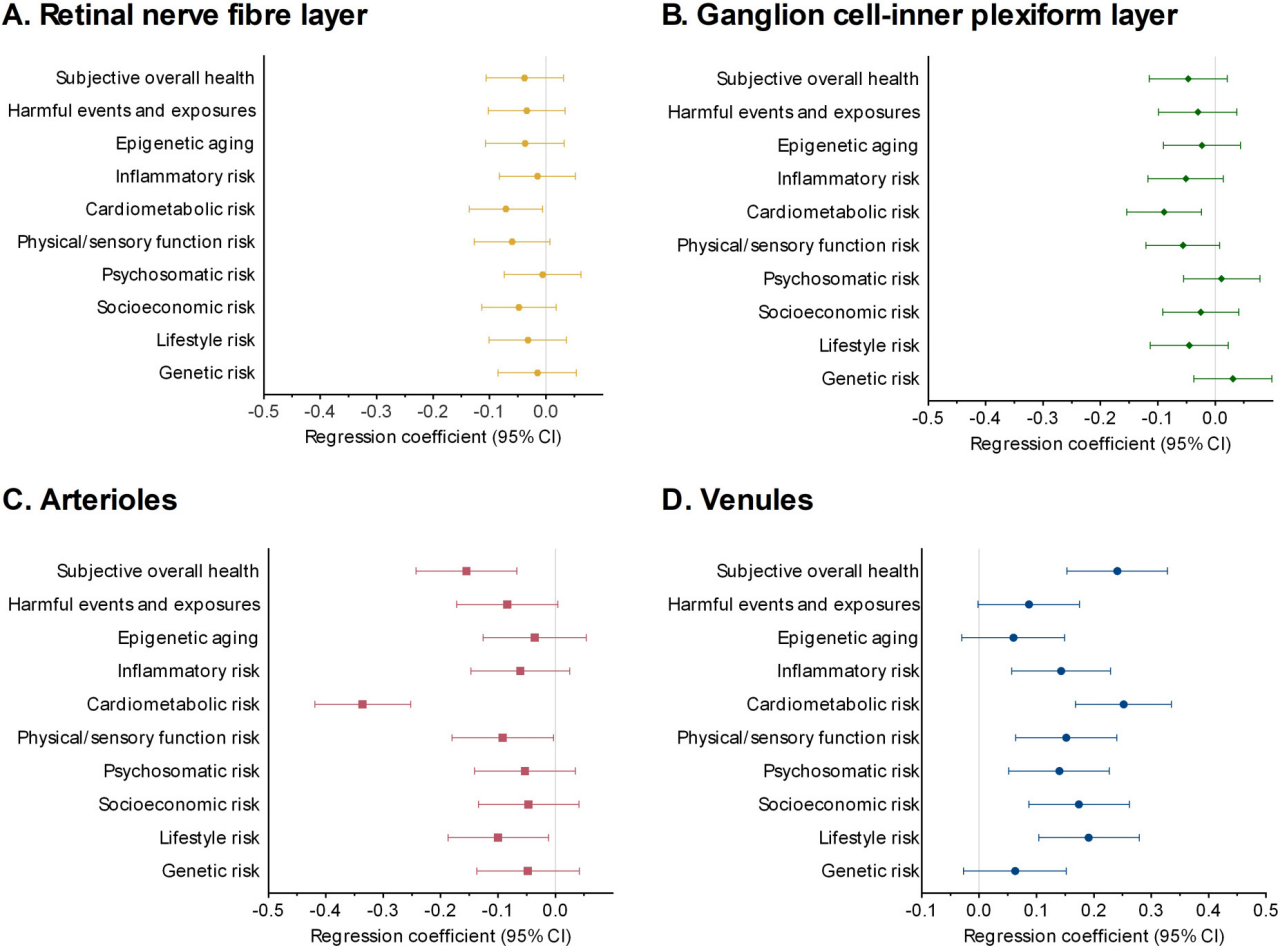
Association of the retinal health measures with the 10 domains of risk comprising the holistic DunedinARB.

As the five ADRD risk indexes capture global ADRD risk across domains as diverse as genetic risk (e.g., *APOE* ε4 status), lifestyle risk (e.g., alcohol consumption, physical activity), cardiometabolic risk (e.g., hypertension, diabetes status), and harmful events (e.g., traumatic brain injuries), the primary analyses may overlook underlying nuanced patterns in the association of retinal health with midlife ADRD risk. Thus, secondary tests investigated the association of the neural and microvascular retinal measures with the 10 domains of risk comprising the holistic DunedinARB (Supplemental Material, Appendix 1). These tests revealed that retinal neuronal measures only captured cardiometabolic ADRD risk (Figure 2) (RNFL β = −0.07 [95%CI −0.14,−0.01], *p *= 0.032, *R*^2 ^= 0.10, not significant after FDR correction; GC-IPL β = −0.09 [−0.15,−0.02], *p *= 0.007, *R*^2 ^= 0.11, remained significant after FDR correction) while the microvascular retinal measures captured many diverse ADRD risk factors. Wider retinal venules, in particular, were associated with greater ADRD risk across 7 out of the 10 measured domains of risk: lifestyle (β = 0.19 [0.10,0.28], *p *< 0.001, *R*^2 ^= 0.04); socioeconomic (β = 0.174[0.09,0.26], *p *< 0.001, *R*^2 ^= 0.03); psychosomatic (β = 0.14 [0.05,0.23], *p *= 0.002, *R*^2 ^= 0.04); physical/sensory function (β = 0.15 [0.06,0.24], *p *= 0.001, *R*^2 ^= 0.03); cardiometabolic (β = 0.25 [0.17,0.34], *p *< 0.001, *R*^2 ^*= *0.15); inflammatory (β = 0.14 [0.06,0.23], *p *= 0.001, *R*^2 ^= 0.06); and subjective overall health (β = 0.24 [0.15,0.33], *p *< 0.001, *R*^2 ^= 0.03). All initially significant associations remained so after FDR correction. Narrower arterioles were associated with greater risk across 4 domains: cardiometabolic (β = −0.34 [−0.42,−0.25, *p < *0.001, *R*^2 ^*= *0.15); lifestyle (β = −0.10 [−0.19,−0.01], *p = *0.03, *R*^2 ^*= *0.04); physical/sensory (β = −0.09 [−0.18,−0.003], *p *= 0.04, *R*^2 ^*= *0.03); and subjective overall health (β = −0.16 [−0.24,−0.07], *p *= 0.001, *R*^2 ^*= *0.03). After FDR correction, associations with cardiometabolic risk and subjective health remained significant.

Figures present standardized regression estimates (βs) and 95% confidence intervals for each retinal measure and the 10 distinct risk domains that comprise the holistic DunedinARB. (A) Retinal nerve fiber layer (RNFL); (B) Ganglion cell-inner plexiform layer (GC-IPL); (C) Arterioles (CRAE); (D) Venules (CRVE).

Post hoc tests adding cardiometabolic risk to the models testing venule diameter associations with the 9 other domains of ADRD risk showed a similar pattern (after FDR correction, associations between CRVE and lifestyle risk, socioeconomic risk, and subjective overall health remained significant; Supplemental Figure 2). This indicates that CRVE was associated with diverse ADRD risk factors regardless of overall cardiometabolic risk.

## Discussion

This observational investigation of retinal health and midlife ADRD risk in a population-representative birth cohort followed to age 45 years produced two findings. First, neuronal and microvascular measures of retinal health were found to distinguish gradients of an individual's midlife risk for later ADRD, as indexed by five dementia risk indexes that capture multifactorial preclinical risk for later dementia. While each dementia risk index selects a different set of risk factors, all have been found to predict later dementia, with some, such as the CAIDE, across 20–40 years of follow-up (C-statistic of 0.75; receiver–operating characteristic [ROC]) of 0.77).^28,29^ The current findings support the hypothesis that measures of retinal integrity could be useful biomarkers for indexing wider ADRD risk, even in midlife. Notably, at this age and in this population, retinal microvascular measures were much more strongly associated with ADRD risk than were retinal neuronal measures. While the retinal nerve fiber layer was found to weakly associate with ADRD risk index scores (and the mean ganglion cell–inner plexiform layer thickness not at all), both arteriole and venule calibers (diameters) were more strongly associated with ADRD risk.

Second, measures of retinal microvascular integrity, particularly venule caliber, were found to associate with all but three domains of ADRD risk (harmful events, genetic, and epigenetic). CRVE, and CRAE to a lesser extent, showed patterns of association across the various domains of ADRD risk, capturing: lifestyle risk factors (physical activity, diet, tobacco smoking, alcohol consumption, folic acid intake, and regular prophylactic NSAID use); physical and sensory function risks (balance, gait, hearing acuity, subjective hearing function, visual contrast sensitivity, subjective visual function, sense of smell); cardiometabolic risk factors (blood pressure/hypertension, BMI, diabetes, total cholesterol and glycerides); and subjective overall health (self-rated, informant-rated, and research worker-rated). Given that the retinal microvascular system is part of the cardiovascular system, and thus subject to the same risk and protective factors, these findings are unsurprising. Interestingly, however, CRVE was associated with additional risk domains less likely to be purely resulting from cardiovascular risk: socioeconomic status (occupational and educational attainment); psychological and somatic function (pain interference, migraine, depression, loneliness/social isolation, sleep quality, and neuroticism & conscientiousness); and inflammatory risk (levels of CRP, IL-6, and suPAR, and rheumatoid arthritis). This final finding reinforces the emerging consensus that wider venule caliber may reflect the influence of chronic inflammation as well as cardiovascular dysfunction.^30^ Both cardiovascular and inflammatory dysfunction are implicated in the etiology of most ADRDs (including Alzheimer's disease, FTD, LBD, MED, and obviously VCID),^9^ although the timing and mechanisms underlying these associations are complex and not fully characterized. In some cases (e.g., chronic neuroinflammation in Alzheimer's disease), dysfunction is believed to precipitate hallmark disease pathology (e.g., the deposition of amyloid beta resulting from increased microglia activation)^31^ and in other cases (e.g., LBD), inflammation is believed to arise from the disease pathology itself (e.g., accumulation of abnormal alpha-synuclein may trigger neuroinflammation).^32^

Interestingly, the measures of retinal *neuronal* integrity were only associated with one domain of ADRD risk: cardiometabolic. This domain encompassed risk factors that spanned individual risk indicators of high body mass index, hypertension, high cholesterol, diabetes, coronary heart disease, and chronic kidney disease.^24^ Both neuronal measures—retinal nerve fiber layer and ganglion cell–inner plexiform layer—are measures of neural thickness; the first a measure of the axons of the retinal ganglion cells at the optic disk (where they leave the retina to form the optic nerve) and the second a measure of the somata and dendrites of the ganglion cells at the macula. These cells are thought to undergo the same pathological processes as the rest of the central nervous system, and thus are thought to be a more direct measure of neuropathology than many other indicators of ADRD risk.^33^ Prior research indicates that these parameters, particularly RNFL, are associated with Alzheimer's dementia and pre-dementia (mild cognitive impairment), although a specific association with vascular dementia has not been reported.^8,11,12,34^ In the current study, a unitary midlife association with cardiometabolic risk suggests two things: first, that retinal ganglion cells are sensitive to the wider body's cardiometabolic state; second, that these might be good biomarkers for early ADRD specifically related to cardiometabolic processes (e.g., vascular dementia pathology) distinct from other processes potentially related to dementia risk (e.g., traumatic brain injuries, loss of sensory function). Conversely, given that the retinal nerve fiber layer has been robustly associated with MCI and dementia diagnoses at later ages,^35–37^ our findings could also suggest that this biomarker may be measuring a component of ADRD risk that is not included in existing ADRD risk indexes. Future research should investigate whether the addition of RNFL or other retinal measures improves the predictive utility of existing ADRD risk indexes.

This study has limitations. First, it was observational and cannot establish causation. Second, it was conducted in one cohort in one country and should be replicated in other settings. The current study cohort was predominantly New Zealand European/Pākehā (White), matching the ethnic composition of the South Island of New Zealand. Evidence that retinal parameters may differ among different racial and ethnic groups indicate that the results should be replicated in other diverse samples.^38,39^ Third, the cohort was not sufficiently aged to have developed ADRDs. Our risk index outcome measures are “highly predictive of the likelihood of dementia decades later” but are not themselves direct measures of dementia pathology or endpoint disease.^28,29^ Future investigation should test the association of midlife retinal health with later ADRD disease diagnoses, onset or progression. Fourth, growing interest in applying artificial intelligence (AI) and deep learning (DL) approaches to retinal imaging holds promise for future research.^40,41^ However, these were not applied in the current study, which focused on testing specific measurable and known biological features in the retina to allow greater insights into the mechanisms underlying the observed associations. Future research could leverage AI and DL alongside specific biological parameters for more exploratory and specific retinal health-outcome testing.

Furthermore, we only removed participants from the analytic dataset if they had disorders that were affecting the retina (e.g., diabetic retinopathy). Any participants with comorbidities such as diabetes or hypertension that may affect retinal measurements were retained in the dataset. The addition of cardiometabolic risk to the models marginally changed our findings: arterioles were no longer associated with other domains of risk, suggesting that CRAE largely captures cardiometabolic risk; while venules remained associated with lifestyle, socioeconomic, and subjective health risk factors, indicating the CRVE uniquely captures aspects of ADRD risk beyond cardiometabolic risk. Future research could investigate whether retinal health measures differentially predict ADRD risk among individuals with specific cardiometabolic comorbidities.

### Conclusion

In this longitudinal observation of a population-representative birth-cohort followed to midlife, widely available, non-invasive, and low-cost measures of retinal health were significantly associated with midlife risk for later ADRD, reflecting most known domains of ADRD risk factors. Digital fundus imagery is an accessible, scalable modality that could provide insights into ADRD disease processes alongside other clinical data, potentially enhancing the ability to predict ADRD risk at the population level long before disease end-points emerge.

## Supplemental Material

sj-docx-1-alz-10.1177_13872877251321114 - Supplemental material for Measures of retinal health successfully capture risk for Alzheimer's disease and related dementias at midlife Supplemental material, sj-docx-1-alz-10.1177_13872877251321114 for Measures of retinal health successfully capture risk for Alzheimer's disease and related dementias at midlife by Ashleigh Barrett-Young, Aaron Reuben, Avshalom Caspi, Kirsten Cheyne, David Ireland, Jesse Kokaua, Sandhya Ramrakha, Yih-Chung Tham, Reremoana Theodore, Graham Wilson, Tien Yin Wong and Terrie Moffitt in Journal of Alzheimer's Disease
